# Rayleigh scatter based order of magnitude increase in distributed temperature and strain sensing by simple UV exposure of optical fibre

**DOI:** 10.1038/srep11177

**Published:** 2015-06-16

**Authors:** Sébastien Loranger, Mathieu Gagné, Victor Lambin-Iezzi, Raman Kashyap

**Affiliations:** 1 Fabulas Laboratory, Engineering Physics Department, Polytechnique Montréal, 2900 boul. Édouard-Montpetit, Montreal H3T 1J4, Canada; 2 Electrical Engineering Department, Polytechnique Montréal, 2900 boul. Édouard-Montpetit, Montreal H3T 1J4, Canada

## Abstract

We present a technique to improve signal strength, and therefore sensitivity in distributed temperature and strain sensing (DTSS) using Frequency domain Rayleigh scatter. A simple UV exposure of a hydrogen loaded standard SMF-28 fibre core is shown to enhance the Rayleigh back-scattered light dramatically by ten-fold, independent of the presence of a Bragg grating, and is therefore created by the UV exposure alone. This increase in Rayleigh back-scatter allows an order-of-magnitude increase in temperature and strain resolution for DTSS compared to un-exposed SMF-28 fibre used as a sensing element. This enhancement in sensitivity is effective for cm range or more sensor gauge length, below which is the theoretical cross-correlation limit. The detection of a 20 mK temperature rise with a spatial resolution of 2 cm is demonstrated. This gain in sensitivity for SMF-28 is compared with a high Ge doped photosensitive fibre with a characteristically high NA. For the latter, the UV enhancement is also present although of lower amplitude, and enables an even lower noise level for sensing, due to the fibre’s intrinsically higher Rayleigh scatter signal.

Optical fibre distributed temperature and strain sensors (DTSS) systems are extremely useful for industrial infrastructure monitoring, since they provide real-time information along a region of interest with low-cost optical fibre. Optical time domain reflectometry (OTDR) with Rayleigh scatter has been used for decades to investigate distributed information along a fibre^1^,^2^. It has been demonstrated for DTSS^3^,^4^ in long lengths of fibre (~km), but with poor spatial resolution (~m) and very poor temperature resolution (~10 °C). On the other hand, its Frequency Domain (OFDR) counterpart gives the highest spatial resolution in DTSS (~mm) while allowing a reasonable temperature resolution (0.1 to 1 °C)^5^ and remains a rather simple and cheap scheme, compared to other DTSS techniques. However, Rayleigh scatter OFDR has remained quite limited in terms of sensing length (30-100m). For this reason, other methods have been developed such as Raman scattering (ROTDR)^6^,^7^, which allows a much longer reach of 1-30 km, or Brillouin scattering (BOTDR or BOTD analysis: BOTDA)^8^,^9^, with an even longer reach of 10–100 km. Both of these techniques however show less advantageous resolution in temperature (~1 °C) and spatially (1–10 m)^10^. Combinations of techniques have also been proposed: Rayleigh and Brillouin scattering, also known as Landau-Placzek ratio analysis^11^, and Rayleigh with Raman scattering^12^.

The main limitation in the sensitivity and accuracy of Rayleigh scatter DTSS comes from the low scattering signal at the detector. Higher scattering medium, such as liquids in hollow core fibres^13^, polymer fibres with larger scattering cross-sections^14^, or specially designed high scattering silica fibres doped with various impurities can be used to increase this signal, thus increase the sensitivity. However such schemes are non-standard and therefore expensive to produce and to render compatible with available optical equipment. We propose and demonstrate here a simple and affordable method to radically improve temperature and strain resolution by an order of magnitude through a dramatic increase in Rayleigh scatter in standard fibre. This increase comes from simply exposing the fibre core to UV light, which creates a high density of scattering defects, such as observed by Johlen *et al.*^15^ in their study of UV exposure induced loss. Such enhancement in fibre can be easily induced with any UV laser (solid state, argon) without any critical alignment or vibration control unlike when writing FBGs. We present a brief overview of Rayleigh OFDR and then present our results showing an order of magnitude improvement in sensitivity for DTSS for standard fibre as well as in a photosensitive high Ge doped core fibre. This increase through UV-exposure is also compared to UV-written long fibre Bragg gratings (FBG) measured *out-of-band*.

## Theory

OFDR allows the measurement of a reflectivity pattern, such as Rayleigh scatter along a fibre length. The Rayleigh back-scatter is caused by defects which induce a local variation in the permittivity. As described by Froggatt *et al.*^16^, such a permittivity variation can be measured with the knowledge of the spectral intensity of an interference between the fibre under test and a reference arm:


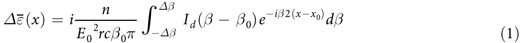


Where *I*_*d*_ is the measured spectral intensity of the interference signal, *n* is the refractive index, *E*_0_ the input laser field, *r* the reflection coefficient of the reference beam and *x*_*0*_ is the position of the reference reflection. Considering that we are working with a system that has discrete sampling, the corresponding integral in Eq. (1), the reflected intensity vs position (therefore in the time domain), can be re-written as:


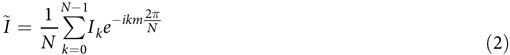


Where *N* is the total number of points within the measurement and *I*_*k*_ is the spectral intensity at different points, *k* along the scan. The measurement of temperature/strain is relative to a reference measurement. Both are compared by doing a cross-correlation over an integration length 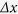, which is called the sensor gauge length and corresponds to the spatial resolution of the DTSS:


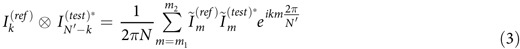


Where *N’* corresponds to the number of points in the integration length 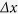 (as 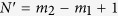), which is considered as the sensor or gauge length, measurement. This cross-correlation is in the frequency domain. When there is no strain or temperature change, a peak is expected at zero frequency. When a temperature or strain change is applied in the sensor gauge length, then this peak shifts proportional to the change. Therefore, the resulting frequency shift is a direct measurement of the observed change in temperature or strain and such a value can be calculated for every sensor gauge lengths,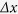. We wish to minimise this length, since it defines the spatial resolution. However, the longer the sensor length, the higher is the peak intensity in the cross-correlation, giving a higher signal to noise ratio (SNR). The noise itself is intrinsic to the calculation and is the nature of Rayleigh scatter, i.e. the random fluctuation in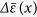, and therefore is always present. This being said, the longer the gauge length, the more precisely can the frequency shift be determined, thus improving the precision of the temperature or strain measurement. The link between frequency shift and strain or temperature is determined by a calibration constant within the OFDR system. It can be shown that such a physical uncertainty limit can be expressed as follows^16^:


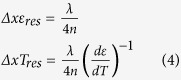


Where 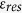 is the strain resolution (dimension-less) and *T*_*res*_ is the temperature resolution. However, with longer lengths of 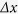
(1 cm or more), another limit appears: the detector intensity noise (not taken into account in Eq. (4)) and the temperature fluctuation along the gauge length.

To improve these limits, we show that a simple UV exposure of the fibre has the effect of increasing the Rayleigh scatter emission and collection by ten-fold, thus generating an increase in temperature and strain resolution. UV exposure of hydrogen (or deuterium) loaded SMF-28, as well as in high germanium content core fibre has the effect of creating color centers^17^,^18^. However, why such exposure would increase Rayleigh scatter, which normally comes from defects in a fibre, is still under investigation. An increase in back-scatter collection can also be accounted for by an increase in the numerical aperture (NA) of the fibre. However, this increase in collected back-scattered power is expected to be limited to a factor of <2, for the expected refractive index change, below 10^−3^ in our demonstration.

## Results

**Figure 1:**
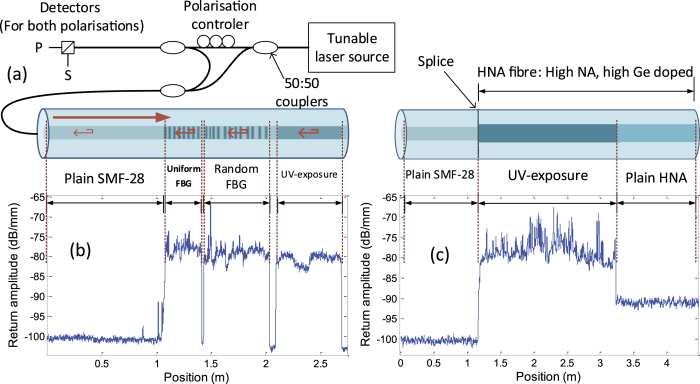
OFDR measurement done with a LUNA system, as shown in (**a**) 20, at different region in the fibre with a wide scan (42 nm). Measurements show the measured round-trip signal amplitude of Rayleigh scatter (corresponds to the square of local losses). (**b**) A comparison of various types of structure and exposure in a standard SMF-28 fibre: uniform FBG, random FBG and continuous UV exposure (no FBG). (**c**) A comparison of SMF-28, with UV-exposed and un-exposed HNA fibre (HNA: High NA fibre with high Ge core). OFDR measurements of gratings were performed *out-of-band* (away from FBG operating wavelength).

Rayleigh back-scatter was measured using a commercial OFDR system from LUNA. This system measures a reflection spectrum with a tuneable scanning laser. Since this is a coherent measurement, a signal will only be measured if the polarisation matches that of the reference. To avoid loss of information from unmatched polarisation, the return signal is compared with two orthogonal polarisations to recover the whole interference spectrum, independently of the fibre’s birefringence, which can also be characterised from this information. The principle is shown in Fig. 1a. A Fourier transform is then performed to recover the temporal (and hence, spatial) reflectivity along the fibre, such as described by Eq. (1). From these results, the temperature and strain can be recovered at any point on the fibre using Eq. (3).

Two types of fibres were tested: a standard SMF-28 single-mode telecom fibre by Corning which was hydrogen loaded for increased photosensitivity and a high NA (0.27) photosensitive fibre with high Ge content from Coreactive (uvs-eps), referred to as HNA in this paper. In the latter fibre, exposure was made by continuously exposing the fibre with UV light, while with the SMF-28, two types of FBG were tested and compared with the continuously UV exposed fibre: a uniform grating and a random grating with randomly positioned phase shifts^19^. FBGs were measured *out-of-band* for DTSS, so as to not be limited by the grating’s bandwidth. Although we expected the FBGs to offer more back-scatter than simple UV exposure since there is more refractive index variation within the fibre, surprisingly, results show that this is not the case, as can be seen in Fig. 1b. Indeed, a continuous UV exposure generates a 20dB increase in Rayleigh back-scatter return signal, which corresponds to a ten-fold increase in Rayleigh-induced local loss, when taking into account the round-trip nature of the measurement which squares the local loss. Back scatter in the presence of the grating, uniform or random, also increases by the same order of magnitude. Although a more complex structure (oscillations) is observed when a random grating is involved, it was noted that this did not lead to any gain in strain or temperature resolution. Indeed, whether a grating is present or not, the average exposure is the same, and an identical gain was observed in terms of distributed temperature or strain noise. However, we suspect there may be a small contribution from the presence of the FBG, although this would require further investigation.

The UV-exposed HNA fibre shows the same improvement in Rayleigh signal, compared to SMF-28, as can be seen in Fig. 1c. However, when comparing the effect of the exposure itself, i.e. the difference in signal between un-exposed and exposed HNA fibre, the gain is not as great since this fibre already has a rather high Rayleigh signature (~ three times that of SMF-28) before UV exposure. This signature can be due to a larger number of fabrication-induced defects as well as from its higher NA, which allows more collection of the Rayleigh back-scatter. Nevertheless, this UV-exposed fibre exhibits a DTSS noise level even more advantageous than UV-exposed SMF-28. The higher scatter noise of the exposed region in the HNA fibre can be due to higher coherent noise from the higher signal and to this fibre’s intrinsic behaviour with UV exposure, leading to more localised defects. Such peculiar behaviour is still under investigation. It should be noted that the HNA fibre was spliced to an SMF-28 fibre for the measurements. Such a splice of two fibres with different mode-field-diameters exhibits some loss, which we estimate to be ~1 dB. This reduction in signal can slightly degrade the performance of the HNA fibre, therefore we can expect a corresponding improvement in the measurement if the splice is optimised (SMF-28 adiabatically tapered to match the mode-field-diameters). However, the unoptimised splice does not have a significant impact on the conclusions of our observations.

**Figure 2:**
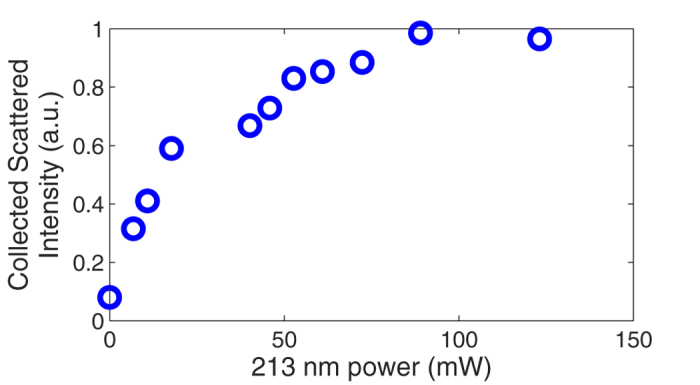
Increase in collected Rayleigh scatter intensity with UV exposure power at constant speed of exposure. These results were taken were averaged for passages at different power levels.

The gain in back-scattered signal gives rise to a considerable increase in temperature and strain sensitivity. Indeed, with this improvement in signal to noise ratio, the cross-correlation of Eq. (3) yields a more precise frequency shift, thus a higher sensitivity in temperature or strain measurement. Note that these measurements are in temperature, but the same applies to strain measurements with a factor of 8.32 με/°C (calibration factor for silica fibre from the LUNA system). The collected Rayleigh back-scatter was measured with varying UV exposure power, as shown in Fig. 2, to understand the optimal exposure to minimise requirements and maximise gain in sensitivity. As can be seen, after a rather linear increase, the gain saturates at around an order-of-magnitude increase. Our choice of power and exposure time for DTSS tests were based on the best exposure conditions. We suspect that this increase in scatter is from the creation of colour centres defect, known to appear for UV exposure as the mechanism for refractive index increase^17^. To increase scatter, these colour centres would have to be highly localised as point defects and exhibit a high scattering cross-section. Saturation could appear as a result of all the available GeO_2_ centres being affected by UV exposure. The reasons for the increase in scatter are still under investigation. A similar rise in scatter and saturation with UV exposure was also observed in other high germania core optical fibre.

**Figure 3:**
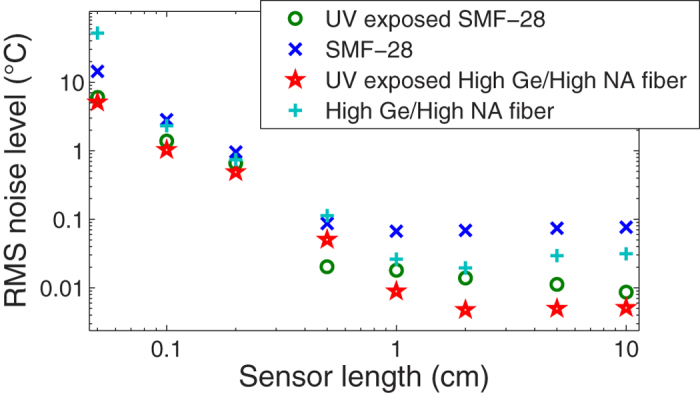
Comparison of noise level (from a section of 30 cm or more) vs sensor length (spatial resolution of DTSS) for exposed (50 mW) and un-exposed SMF-28 and HNA fibre (High NA with high Ge core). The UV enhancement can be seen for a sensor length of more than 1 cm. For these measurements, the sensing fibres were housed in a thermally stable environment (insulated container) to avoid thermal fluctuations to observe the measurement noise alone.

A quantitative analysis is shown in Fig. 3 where the RMS noise level was calculated based on a 30 cm section of 1 mm spaced points. The sensor cross-correlation integration length, which defines the spatial resolution of the DTSS, was varied from a long length of 10 cm to a very short length of 1 mm. The limit defined by Eq. (4) can be observed in these results for short sensor lengths. The higher SNR of the UV exposed fibre seems to slightly improve the resolution within this range, where the noise is limited by the cross-correlation on a random structure, typical of Rayleigh scatter. The most important gain is in the cm range, where the detection noise, unrelated to back-scatter from the material structure and defects, becomes the dominating limitation. In this range, the noise level can be expected to rise slightly as the sensor length increases, since it becomes more sensitive to thermal fluctuation along its length. However, the sensing fibre tested here was in a thermally stable isolated container, which explains why the thermal noise is more stable and actually decreases slightly with sensor length. This shows the performance limit of the DTSS system itself, independent of the environment. From the results in Fig. 3, we show an RMS noise level of 10–15 mK in temperature for gauge lengths of 2 cm or more for UV-exposed SMF-28. If converted to strain, this results in an RMS noise level of 80–120 nε. The UV-exposed HNA fibre’s performance is twice as good compared to the SMF-28 with an RMS noise level of ~5 mK (40 nε), the best performance to date with this resolution level.

**Figure 4:**
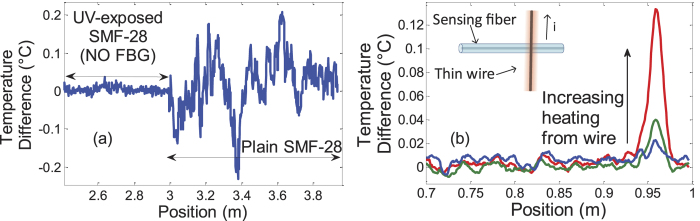
Examples of distributed measurements of temperature change. (**a**) for a uniform temperature along the fibre (in a thermally stable and insulated container), the difference in noise between UV-exposed and un-exposed SMF-28 can be observed clearly. (**b**) For a local heating done by a thin wire (0.2 mm in diameter), we can observe the point spread function of the distributed sensor (spatial resolution) in UV-enhanced fibre. The curves (blue to red) are from an increasing current on the thin wire. As shown, a heating of 20 mK can be detected by such a distributed sensor. The sensor integration length was 2 cm, which is also shown by the width of the point spread function. A spatial averaging of surrounding points (1 cm wide) was performed to further reduce noise.

The difference in noise and ease of measurement can be appreciated in Fig. 4a, where we can clearly see the improvement in the measurement along the fibre, which is placed in an isothermal, stable and isolated container. In the latter, the effect of the UV-enhancement is clearly seen, where temperatures in the 10–100 mK range can be resolved, while this is impossible in the non-exposed fibre area, where the noise level is much higher. Another measurement example is shown in Fig. 4b where the spatial and temperature resolution can be appreciated. In this case, a thin 0.2 mm diameter wire heated by a low current of 20 to 100 mA is placed in contact with the fibre (UV-exposed SMF-28) in a perpendicular fashion (shown in the inset in Fig. 4b). With a 2 cm sensor integration length, the point-spread-function of such a DTSS measurement is observed. The heating by 20 mK is seen in the bottom curve in Fig. 4b (blue curve). To further increase the resolution and measurement quality, a spatial averaging was performed on the surrounding points. The equivalent length of this spatial averaging was chosen as half the sensor integration gauge length so as to not further limit the spatial resolution.

## Discussion

While it has been nominally noted in the past^15^ that UV exposure increases back scatter in optical fibre, there has been no systematic study to either understand it or to apply it. In this paper we have undertaken to find the conditions to maximise Rayleigh scatter through UV irradiation and then to use the increase in what we believe is the first application in sensing.

We have shown here that a simple continuous UV exposure of a hydrogen loaded SMF-28, increases the back-scattered Rayleigh intensity by ten-fold, thus allowing a ten-fold increase in DTSS sensitivity. Increase in the collected back-scatter from a gain in the NA of the fibre can account for a factor of only 2 for the exposure used here, therefore the remaining back-scatter signal gain comes from an increase in Rayleigh scatter itself. The reason for this increase is still under investigation. The increase in the signal, greatly improves the SNR at the detector, therefore improving the noise floor in DTSS measurements to the theoretical sensitivity/spatial resolution limit. Indeed, for sensor integration lengths of 2 to 10 cm, an RMS noise level of 10 mK or 80 nε was obtained in a thermally stable environment in standard UV exposed H_2_ loaded fiber, after the removal of the hydrogen. The improvement in Rayleigh scatter is not related to the hydrogen as it was allowed to diffuse out completely before measurements. An even lower noise floor was shown with a high NA photosensitive fiber to 5 mK or 40 nε, the best reported performance, to our knowledge, for a 1–2 cm range gauge length. In comparison, Gifford *et al.* demonstrated recently a 1 mK resolution, but with a 12 cm gauge length using weak semi-continuous FBGs to increase return signal^20^. However, with the use of in-band signals from Bragg gratings, one is limited by the bandwidth of the grating, thus limiting the spatial resolution. Since UV-exposure affects the entire spectrum of Rayleigh scatter, our method of improvement does not involve any theoretical bandwidth limit, except equipment limitation from the scan range and practical consideration of the measurement time.

With a saturating exposure in standard fibre, we can expect to further push back this limit to 5 mK noise level for a 2 cm sensor and perhaps 1 mK for a 10 cm spatial resolution. Although such a sensor would be comparable to the one in reference 20, it remains simpler and easier to fabricate, since no holographic FBG inscription equipment is required. Improving back-scatter is very simple, since it only requires a UV laser and a focusing element. No critical alignment or vibration stabilization is required. Although we used hydrogen loading of SMF-28 to improve photosensitivity in our demonstration, the same effect was shown in photosensitive fibre exhibiting a similar UV interaction mechanism, i.e. color center generation, such as in highly germania-doped fibre. Therefore, UV exposure can be performed easily during the drawing process in such a photosensitive fibre before the coating phase. It is also a much easier technique to improve sensitivity than writing multiple FBGs along the sensing fibre^20^, which also improves sensitivity, while sacrificing spatial resolution and increases fabrication complexity. It should be noted that this improvement can only benefit the temperature/strain resolution and spatial resolution. The maximum sensing range is limited by the minimum step of the laser scan through the Fourier transform, although different spatial position can be probed by changing the reference arm length. However, the same UV enhancement can be applied to coherent OTDR, which only takes more time for a measurement, where the equipment limits spatial resolution but not the maximum sensing length.

## Method

Continuous UV exposure was performed using our high precision fibre Bragg grating (FBG) writing system without writing an FBG. The fibre core was illuminated with 213 nm wavelength from the 5^th^ harmonic of a 1064 nm solid-state laser (from Xiton Photonics GmbH). For DTSS tests, the fibre was exposed with 50 mW of power at 50 μm per second with a spot size of ~200 μm giving a uniform exposure time of ~4 seconds. Continuous UV exposure was compared to an *out of band* OFDR signature of FBGs written with the same exposure time and power, by a direct holographic writing technique^21^ using the same experimental setup. For scattering characterisation tests with exposure, power or speed was varied along a length of 100 mm. DTSS was performed using a commercial OFDR system from LUNA^22^. Cross-correlation to resolve the frequency shift was also performed by the same commercial system. The sensing fibre was placed in a thermally stable environment (insulated box) to eliminate thermal fluctuations to provide a real sense of the measurement’s noise limits. The long gratings were interrogated *out-of-band* to allow maximum penetration of the input light and to ensure a maximum sweeping range of the OFDR system. In such a case, it is not the grating resonance that is used, but the microscopic index variation due to the periodic nature of the refractive index modulation and which might generate enhanced back-scatter. The measurements take around 5 s for a 40 nm bandwidth scan plus a few seconds for the temperature or strain calculation. In order to ensure there is no contribution to the Rayleigh scatter measurements from the molecular hydrogen in the fibre, all measurements were performed in the weeks following the UV exposure, to allow the hydrogen to diffuse out at room temperature.

## Additional Information

**How to cite this article**: Loranger, S. *et al.* Rayleigh scatter based order of magnitude increase in distributed temperature and strain sensing by simple UV exposure of optical fibre. *Sci. Rep.* **5**, 11177; doi: 10.1038/srep11177 (2015).
